# The universe of brief psychosis

**DOI:** 10.1192/j.eurpsy.2021.1377

**Published:** 2021-08-13

**Authors:** T. Coelho Rocha, J. Cunha, S. Torres, A. Lopes

**Affiliations:** Psychiatry And Mental Health Department, Centro Hospitalar Barreiro-Montijo, Barreiro, Portugal

**Keywords:** Bouffee Delirante, Brief psychosis, Psychotic disorders, Atypical psychosis

## Abstract

**Introduction:**

Nowadays, ‘Acute and transient psychotic disorders’ in ICD-10 and ‘Brief psychotic disorders’ in DSM-5 are both classifications of the same clinical entity. Over the years, several concepts have been formulated to define the same syndrome.

**Objectives:**

To explore the historical evolution of brief psychotic disorders and relate them to current nosologies.

**Methods:**

Literature review, using the most relevant papers, with the keywords “brief psychosis”, “bouffée délirante”, “cycloid psychosis”, “psychogenic psychosis”, “atypical psychosis” and “holodysphrenia”.

**Results:**

Initially, in 1896, Kahlbaum coined the term ‘dysphrenia’, a group of severe form of psychosis that remitted without showing the typical sequence of disease states and without leaving a lasting alteration. Later, Kraepelin included this kind of disorder in manic depressive illness, which he first named as ‘periodic delirium’ and then as ‘delirious mania’. Magnan, in the pre-Kraepelinian era, created the term ‘bouffée délirante’, a sudden onset of delusional ideas with rapid evolution and intense symptomatology with complete remission usually followed after a short time. Later on, Henry Ey grabbed this entity and renewed it, contrasting it to the defined concept of schizophrenia. Other psychiatric schools have proposed numerous designations: ‘cycloid psychosis’ by Kleist from the German school, ‘psychogenic psychosis’ by Wimmer of the Scandinavian school and ‘holodysphrenias’ by Barahona-Fernandes from the Portuguese school. Cultural variants are also observed, as ‘amok’ seen in Malaysia or ‘shinbyung’ in Korea.

**Conclusions:**

The intensity and polymorphism of brief psychosis present a clinical challenge. The historical evolution may be helpful on recognizing this entity in current clinical practice.

